# Strategies of Detecting Bacteria Using Fluorescence-Based Dyes

**DOI:** 10.3389/fchem.2021.743923

**Published:** 2021-08-12

**Authors:** Shin A Yoon, Sun Young Park, Yujin Cha, Lavanya Gopala, Min Hee Lee

**Affiliations:** Department of Chemistry, Sookmyung Women’s University, Seoul, South Korea

**Keywords:** bacteria detection, lipopolysaccharide, nitroreductase, alkaline phosphatase, β-lactamase, caspase-1, peptidoglycan synthesis, photodynamic therapy

## Abstract

Identification of bacterial strains is critical for the theranostics of bacterial infections and the development of antibiotics. Many organic fluorescent probes have been developed to overcome the limitations of conventional detection methods. These probes can detect bacteria with “off-on” fluorescence change, which enables the real-time imaging and quantitative analysis of bacteria *in vitro* and *in vivo*. In this review, we outline recent advances in the development of fluorescence-based dyes capable of detecting bacteria. Detection strategies are described, including specific interactions with bacterial cell wall components, bacterial and intracellular enzyme reactions, and peptidoglycan synthesis reactions. These include theranostic probes that allow simultaneous bacterial detection and photodynamic antimicrobial effects. Some examples of other miscellaneous detections in bacteria have also been described. In addition, this review demonstrates the validation of these fluorescent probes using a variety of biological models such as gram-negative and -positive bacteria, antibiotic-resistant bacteria, infected cancer cells, tumor-bearing, and infected mice. Prospects for future research are outlined by presenting the importance of effective *in vitro* and *in vivo* detection of bacteria and development of antimicrobial agents.

## Introduction

Bacteria are small single cell organisms and millions of bacteria exist in the human body. Most bacteria are harmless to humans and play an important role in the digestive tract, vitamin production, and destruction of diseased cells (Zhang et al., 2015). However, some bacteria can cause serious infections and even death. For example, the World Health Organization (WHO) reported some of the pathogens that may cause the greatest harm to human health (Hu et al., 2018). Moreover, multidrug-resistant pathogenic bacteria are emerging as a serious problem worldwide due to the abuse of antibiotics (Jeong et al., 2016). Thus, the identification of bacterial strains is critical for the treatment of bacterial infections and the development of antibiotics.

Pathogenic bacteria are classified into two categories according to their cell surface composition: Gram-positive and Gram-negative (Scott and Barnett, 2006; Hanassi et al., 2012; Mai-Prochnow et al., 2016). Gram-positive bacteria are characterized by the presence of thicker, more cross-linked peptidoglycans and no outer membrane (Scott and Barnett, 2006). However, Gram-negative bacteria have a thin layer of peptidoglycan (PG) wall and an outer membrane consisting of lipoproteins, such as phospholipids and lipopolysaccharide (LPS) (Hanassi et al., 2012). On the other hand, various bacterial enzymes are involved in the biosynthesis of bacterial cell walls, nucleic acids, and metabolites and are closely related to the bacterial infection process (Bouhss et al., 2008; Egorov et al., 2018). It is also known that bacterial resistance is associated with structural changes in bacterial enzymes (Egorov et al., 2018). For instance, nitroreductase (NTR), a family of flavin-containing enzymes, is widely expressed in Gram-positive and Gram-negative bacteria. NTR converts aromatic nitro groups to amines using NADH as a cofactor (Roldan et al., 2008). In addition, *E. coli* has been identified as a high-expressing strain of alkaline phosphatase (ALP) involved in dephosphorylation (Kantrowitz, 2011). Antibiotic-resistant bacteria also express high levels of *β*-lactamases (Shapiro, 2016; Cheng et al., 2018). Moreover, some enzymes, such as caspase-1, are also activated when human cells are infected (Sokolovska et al., 2013).

Various techniques have been used for bacterial detection, such as polymerase chain reaction (PCR), gram-staining, immunological techniques, and Raman spectroscopy (Lazcka et al., 2007; Váradi et al., 2017; Hameed et al., 2018; Franco-Duarte et al., 2019; Yoon et al., 2021) For example, PCR methods use amplified nucleic acids to identify DNA sequences, immunological techniques utilize the antibody and antigen and Raman spectroscopy can distinguish the bacteria based on difference of light-scattering. However, these methods are often time-consuming, expensive, require complex procedures, and can lead to false positive results.

To overcome these problems, research on the development of fluorescence-based dyes that can discriminate bacteria has been pursued (Huang et al., 2021; Yoon et al., 2021). Fluorescence-based dyes are small organic probes that can provide rapid detection of target analytes with a high selectivity and sensitivity, low cost and ease of operation, wide range of applications in various analytical conditions (Lee et al., 2015; Park et al., 2021). Ideally, fluorescence probes enable real-time imaging of bacteria and quantification of bacteria *in vitro* or *in vivo*. Most probes currently developed are capable of generating “off-on” fluorescence through bacterial-specific reactions and interactions. In addition, internal charge transfer (ICT), twisted intramolecular charge transfer (TICT) and aggregation-induced emission (AIE) have been used as the working principle of fluorescent probes (Hong et al., 2011; Sasaki et al., 2016; Misra and Bhattacharyya, 2018). TICT and ICT are characteristic fluorescence signals induced upon photoexcitation in fluorescent molecules composed of electron donor and acceptor groups. On the other hands, in the AIE phenomenon, AIEgens are non-fluorescent in dissolving conditions due to the free rotation of benzenes causing non-radiative decay, whereas fluorescence emission is greatly increased in aggregating conditions through restriction of free rotation (Mei et al., 2015; Sasaki et al., 2016). More recently, theranostic probes have emerged that can not only analyze bacteria, but also act as photosensitizers that generate reactive oxygen species (ROS) under light irradiation, providing a photodynamic antimicrobial effect.

**FIGURE 1:**
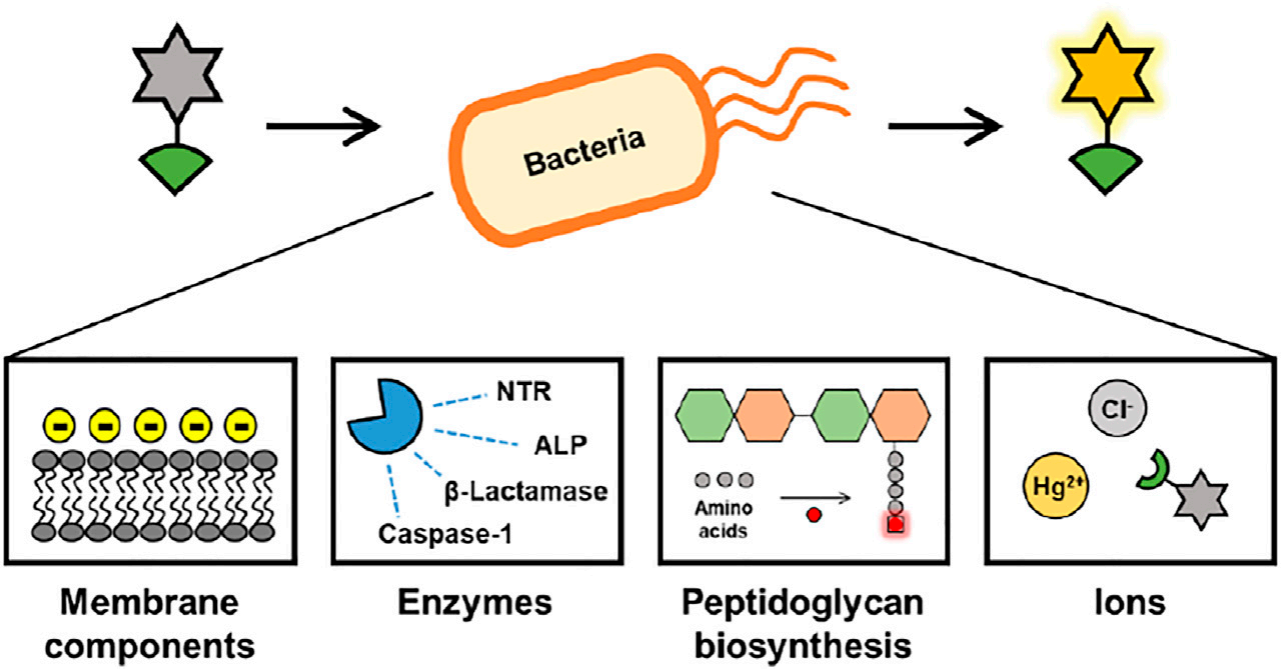
Overview of this review.

In this review, we summarize recent advances achieved in the field of fluorescent probes for a variety of bacteria (Figure 1). The strategy for fluorescent probes targeting bacteria is as follows: 1) detect bacterial membranes based on interactions with cell wall components such as lipopolysaccharide (LPS); 2) detect bacterial enzymes such as nitroreductase (NTR), alkaline phosphatase (ALP), *β*-lactamases, and caspase-1; 3) label chemically fluorescent bacteria using peptidoglycan (PG) synthesis in bacterial cell walls; and 4) detect Hg (II) and chloride in bacteria. Emphasis is placed on the chemical characterization of advanced probes and their use in appropriate applications.

## Fluorescent Probes to Detect Bacterial Membranes

Pathogenic bacteria are classified as Gram-positive or Gram-negative according to their cell surface composition (Mai-Prochnow et al., 2016). Gram-positive bacteria have a relatively thicker cell wall, with more cross-linked peptidoglycans and no outer membrane, whereas Gram-negative bacteria have a thin layer of peptidoglycans as their cell wall and an outer membrane composed of lipoproteins, phospholipids, and lipopolysaccharide (LPS) (Scott and Barnett, 2006; Hanassi et al., 2012). Among them, LPS, known as a cell endotoxin, is a major component of the outer membrane of most Gram-negative bacteria (Bertani and Ruiz, 2018). LPS is crucial for bacterial motility, intestinal colonization, biofilm formation, and antibiotic resistance (Huszczynski et al., 2019). However, the release of large amounts of LPS from Gram-negative bacteria can cause diarrhea, sepsis, and septic shock, which are the leading causes of death (Farhana and Khan, 2020). Recently, advanced research on the development of fluorescent organic probes that can discriminate bacteria by utilizing the physical and chemical properties of bacterial cell walls has been pursued. Fluorescent organic probes allow for real-time imaging of bacteria and quantification of bacteria *in vitro* or *in vivo*.

### Lipopolysaccharide-Based Bacterial Membrane Detection

**FIGURE 2:**
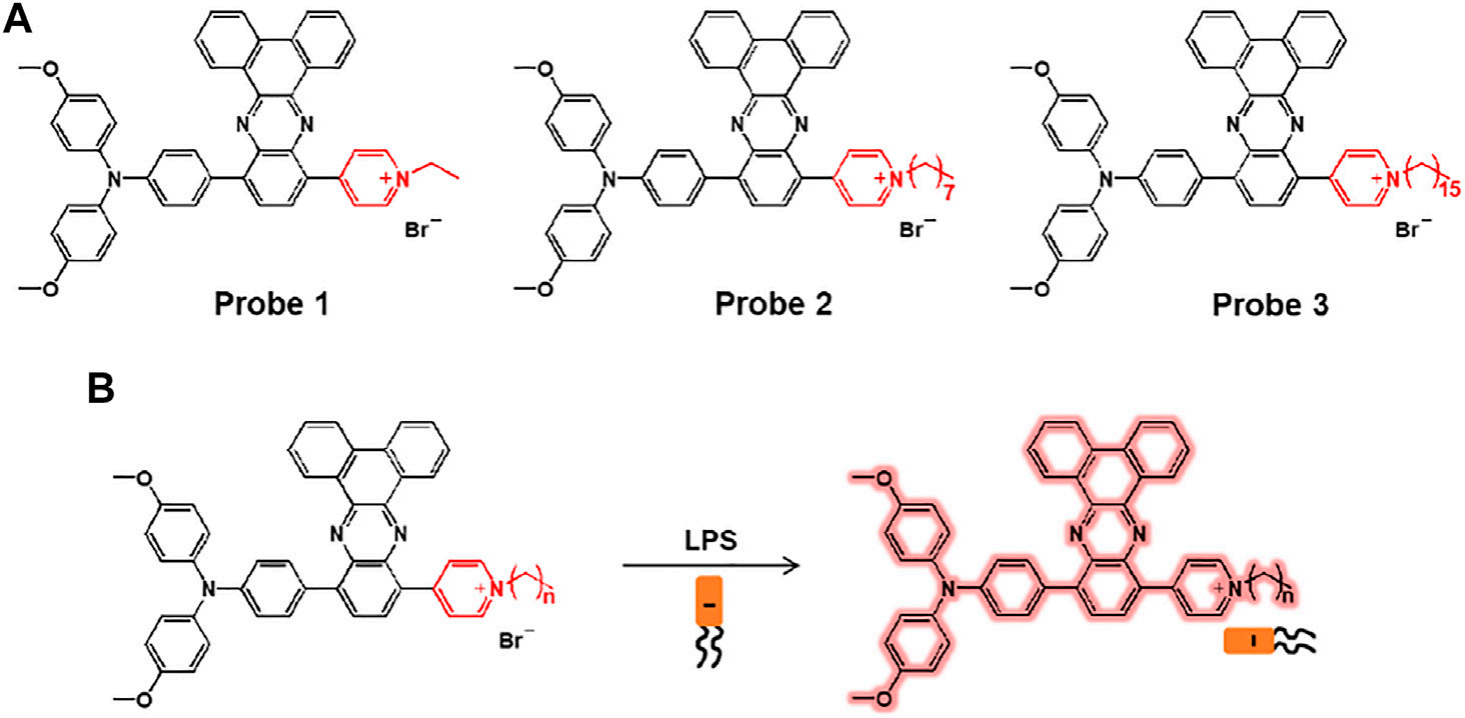
**(A)** Chemical structures of probes 1–3. **(B)** Sensing mechanisms of probes for LPS.

Hua et al. reported a series of lipopolysaccharide (LPS)-selective and reactive oxygen species (ROS) producible fluorescent probes 1–3 composed of pyridinium-functionalized dibenzo[a,c]phenazines with alkyl chains of varying lengths, ethyl, octyl, and hexadecyl (Figure 2) (Liu et al., 2019). The pyridinium salt unit interacts with the negatively charged LPS, which limits the free rotation of the fluorophore, producing the aggregation-induced emission (AIE). The probe showed a maximum absorption peak at around 532 nm and AIE around 720 nm with increasing water fraction. When comparing the probes, probe 1, with a relatively short ethyl chain, showed a significant increase in the fluorescence of LPS. The detection limit of probe 1 for LPS was 0.26 nM. In addition, the singlet oxygen quantum yield of probe 1 was 70.6%, which was superior to that of other probes (probe 2 = 30.7%; probe 3 = 30.2%). In the bio-experiment, probe 1 showed bright fluorescence that was highly sensitive to LPS in *Escherichia coli* (*E. coli*), which is a Gram-negative bacterium. In addition, it was demonstrated that probe 1 acted as an antibacterial photosensitizer under 530 nm laser irradiation to induce photo-induced bacterial death.

**FIGURE 3:**
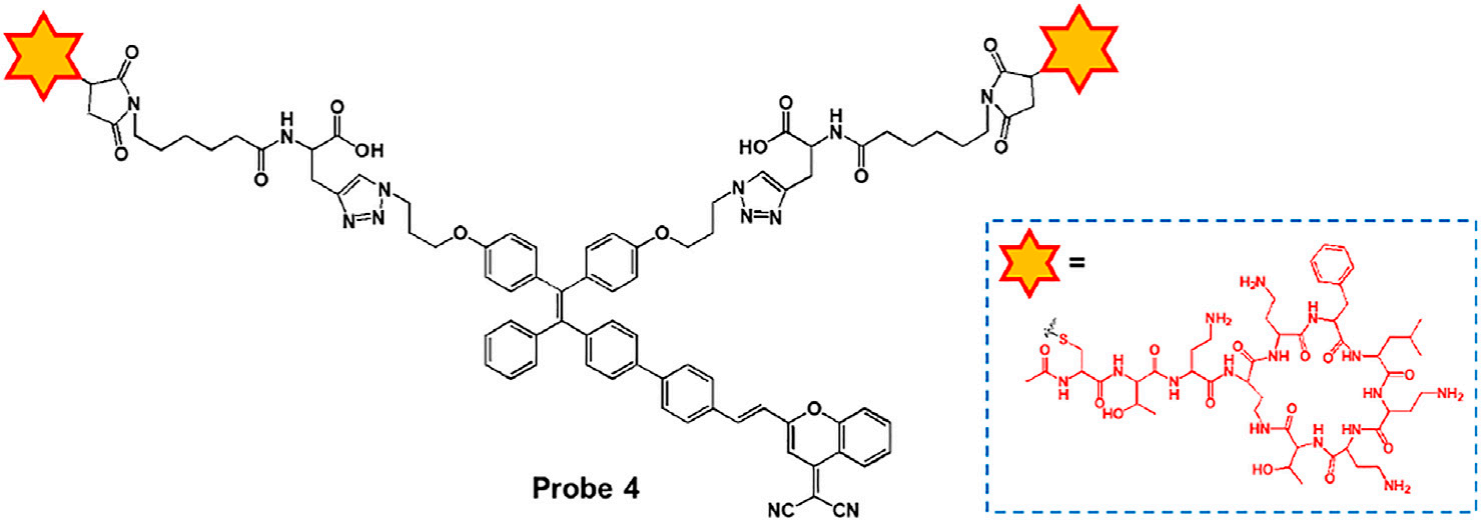
Chemical structure of probe 4.

Ding and co-workers reported the peptide-based aggregation-induced emission (AIE) luminogen, probe 4, for the selective detection of LPS and the photodynamic killing effect on Gram-negative bacteria (Figure 3) (Bao et al., 2021). Probe 4 consists of AIE-DCM for AIE observation and two polymyxin B for specific binding to LPS. Due to the strong interaction between polymyxin B and LPS, the free rotation of AIE luminogen can be inhibited to cause fluorescence enhancement. Probe 4 displayed an absorption at 439 nm and a fluorescence feature at around 650 nm. Upon incubation with Gram-negative bacteria (*E. coli* and *S. enteritidis*) and Gram-positive bacteria (*E. faecalis* and *S. mutans*), probe 4 showed a bright fluorescence feature in the case of Gram-negative bacteria. In particular, the Gram-negative bacteria were killed by specific light irradiation due to the photodynamic antimicrobial effect of probe 4. These results suggest that probe 4 could be a useful tool for detecting Gram-negative bacteria while providing antibacterial effects based on photodynamic therapy (PDT).

### Interaction With Other Components in Bacterial Membrane

**FIGURE 4:**
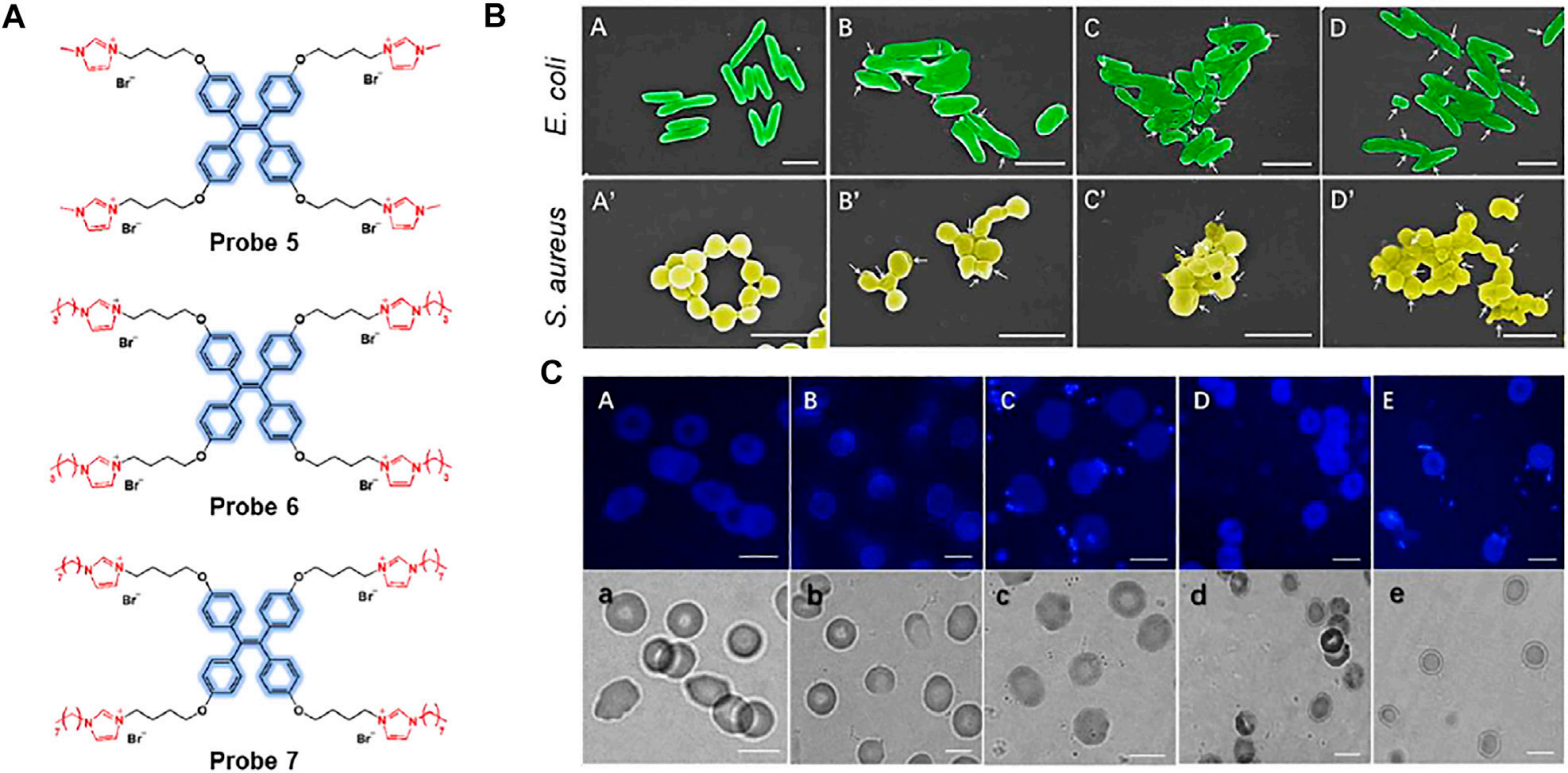
**(A)** Chemical structures of probes 5–7. **(B)** Scanning electron microscopy (SEM) images of *E. coli* and *S. aureus* cultured on polyethylene terephthalate (PET) membranes. **(A**,**A’)** PET, **(B**,**B’)** probe 5, **(C**,**C’)** probe 6, and **(D**,**D’)** probe 7. White arrows indicate collapses and fusion of bacterial cell membranes on the poly(ionic liquid) (PIL) membranes. **(C)** Fluorescence and bright-field images; **(A**,**a)** red blood cells without bacteria, **(B**,**b)** *S. aureus* in red blood cell suspension, **(C**,**c)** *S. aureus* in red blood cell suspension with probe 5, **(D**,**d)** *E. coli* in red blood cell suspension, **(E**,**e)** *E. coli* in red blood cells with probe 5. Reproduced with permission from Shi et al. (2019). Copyright ^©^ 2019, Elsevier.

Yan et al. reported a series of aggregation-induced emission (AIE)-based imidazolium ionic liquids, probes 5–7, for imaging and killing bacteria (Figure 4A) (Shi et al., 2019). The probes consisted of tetraphenylethylene (TPE) as an AIE luminogen and alkyl chains of various lengths (*n* = 1, 4, 8). Cationic imidazolium can detect and kill bacteria via electrostatic and hydrophobic interactions with negatively charged bacterial cells. Upon binding the cationic units of the probes with bacteria surface, the AIE luminogen can emit the fluorescence. The absorption maximum appeared at 258 nm and the fluorescence intensity increased at 475 nm. As shown in Figure 4B, the probes were able to image Gram-negative *E. coli* and Gram-positive *S. aureus*, and showed an effective antibacterial effect as the length of the alkyl chain increased. It was also suggested that this system can be used to kill bacteria and track their viability, as the probes can selectively detect dead bacteria. Furthermore, after incubation with red blood cells and bacteria, the probe could clearly identify *E. coli* and *S. aureus* without affecting red blood cells (Figure 4C).

**FIGURE 5:**
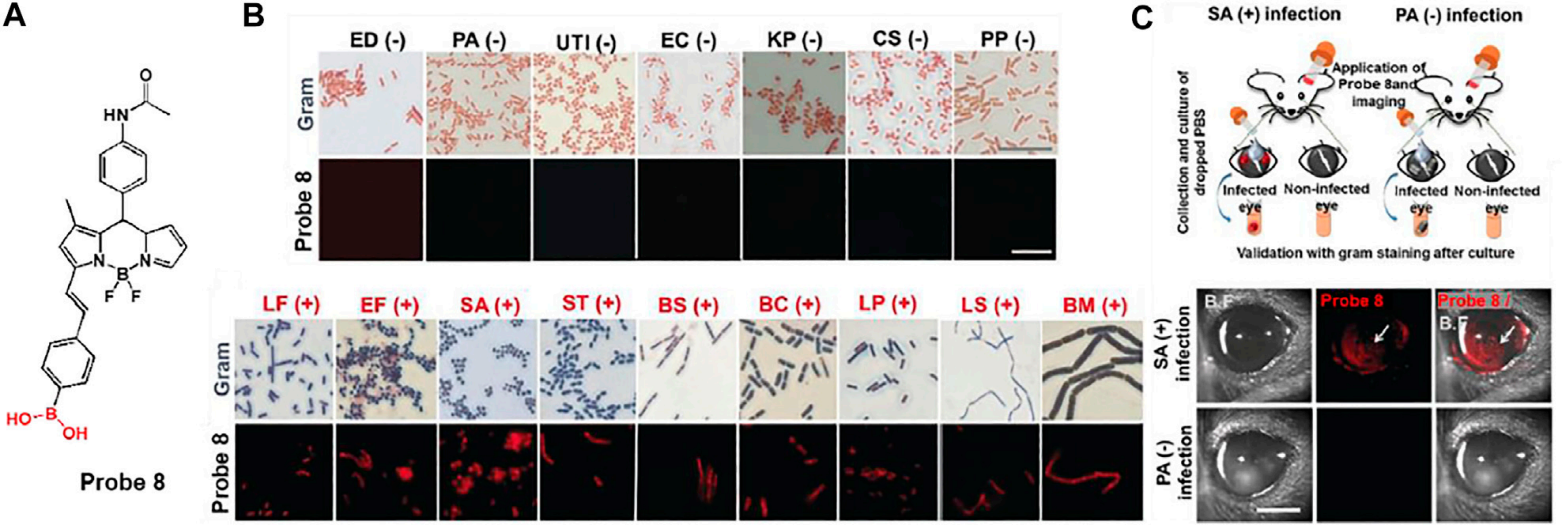
**(A)** Chemical structure of probe 8. **(B)** Gram staining and fluorescence-based staining of 16 bacterial strains. (BC, *Bacillus cereus*; BM, *Bacillus megaterium*; BS, *Bacillus subtilis*; CS; *Cronobacter sakazakii*; EC, *Escherichia coli*; ED, *Escherichia coli DH5a*; UTI, *Escherichia coli UTI89*; EF, *Enterococcus faecalis*; KP, *Klebsiella pneumoniae*; LF, *Lactobacillus fermentum*; LP, *Lactobacillus plantarum*; LS, *Lactobacillus sakei*; PA, *Pseudomonas aeruginosa*; PP, *Pseudomonas putida*; SA, *Staphylococcus aureus*; ST, *Streptococcus thermophilus*) **(C)** Fluorescence imaging in eye-infection model. Reproduced with permission from Kwon et al. (2019). Copyright ^©^ 2019, Wiley-VCH Verlag GmbH & Co. KGaA, Weinheim.

Meanwhile, Chang and co-workers developed BODIPY possessing boronic acid, probe 8, for the selective and sensitive detection of Gram-positive bacteria (Figure 5A) (Kwon et al., 2019). The boronic acid moiety can bind to the glycoprotein structure, which is expressed on the cell surface of Gram-positive bacteria. Through the disaggregation-induced emission effect, probe 8 has a low fluorescence background under aggregation conditions, but shows fluorescence-on upon binding to Gram-positive bacteria (Zhai et al., 2014). In Figure 5B, probe 8 showed a significant fluorescence to various Gram-positive bacteria such as *Lactobacillus fermentum* (LF), *Enterococcus faecalis* (EF), *Staphylococcus aureus* (SA), *Streptococcus thermophilus* (ST), *Bacillus subtilis* (BS), *Bacillus cereus* (BC), *Lactobacillus plantarum* (LP), *Lactobacillus sakei* (LS), and *Bacillus megaterium* (BM), in contrast to Gram-negative bacteria. In addition, a mouse model in which each eye was infected with Gram-negative *Pseudomonas aeruginosa* (PA) and Gram-positive *Staphylococcus aureus* (SA), respectively, showed that probe 8 selectively imaged SA-infected eyes with bright fluorescence as opposed to the case of PA (Figure 5C). From these results, this probe has proven its potential for use in clinical applications for the detection of Gram-positive bacteria.

**FIGURE 6:**
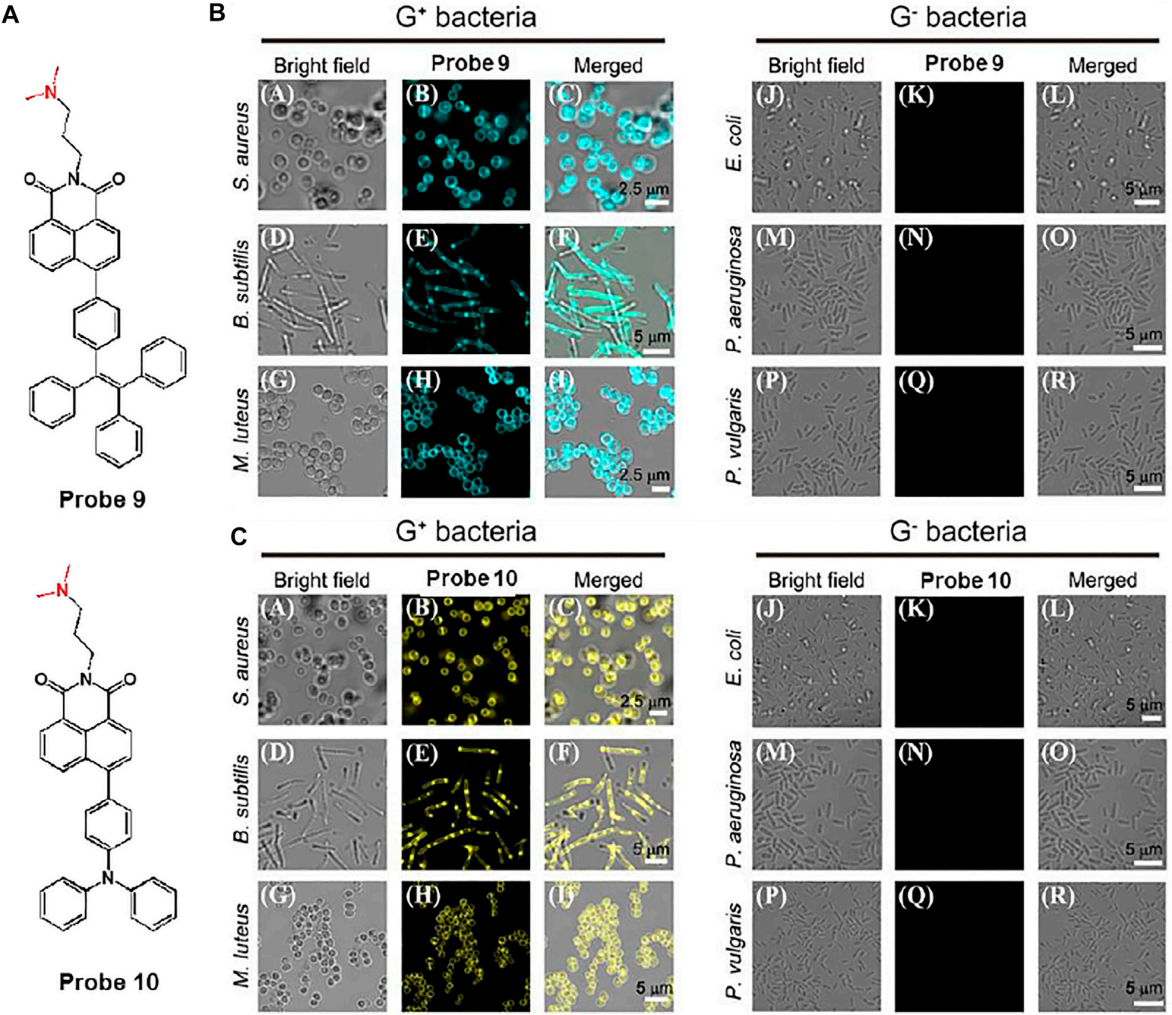
**(A)** Chemical structures of probes 9 and 10. Confocal microscopic images of Gram-positive (G^+^) bacteria and Gram-negative (G^−^) bacteria with the treatment of **(B)** probes 9 and **(C)** 10, respectively. Reproduced with permission from Sayed et al. (2021). Copyright ^©^ 2020, Elsevier.

In another study, Gram-positive bacteria selective aggregation-induced emission (AIE)-based fluorescent probes, probes 9 and 10, were exploited by Lu et al. (Sayed et al., 2021). The fluorescent probes are consisted of naphthalimide as an electron-withdrawing group and tetraphenylethylene (TPE) or triphenlyamine (TPA) as an electron-donating group (Figure 6A). Tertiary amines were introduced for selective targeting because they can interact with lipoteichoic acid and teicuronic acid present on the surface of Gram-positive bacteria. AIE signals of probes was activated after binding to Gram-positive bacteria based on electrostatic and hydrophobic interactions. The absorption/emission maxima of probes 9 and 10 were recorded at 375/537 nm and 430/549 nm, respectively. It was also found that the fluorescence intensity of the probe increased at high proportions of water owing to the AIE effect. In the bacterial staining experiments, the probes could selectively and sensitively stain Gram-positive bacteria such as *S. aureus*, *B. subtilis*, and *M. luteus*, even when Gram-negative bacteria and fungi coexisted (Figures 6B,C). Therefore, it was proposed that these probes could be utilized for gram-type differentiation and could be expanded to AIE-based theragnostic applications.

**FIGURE 7:**
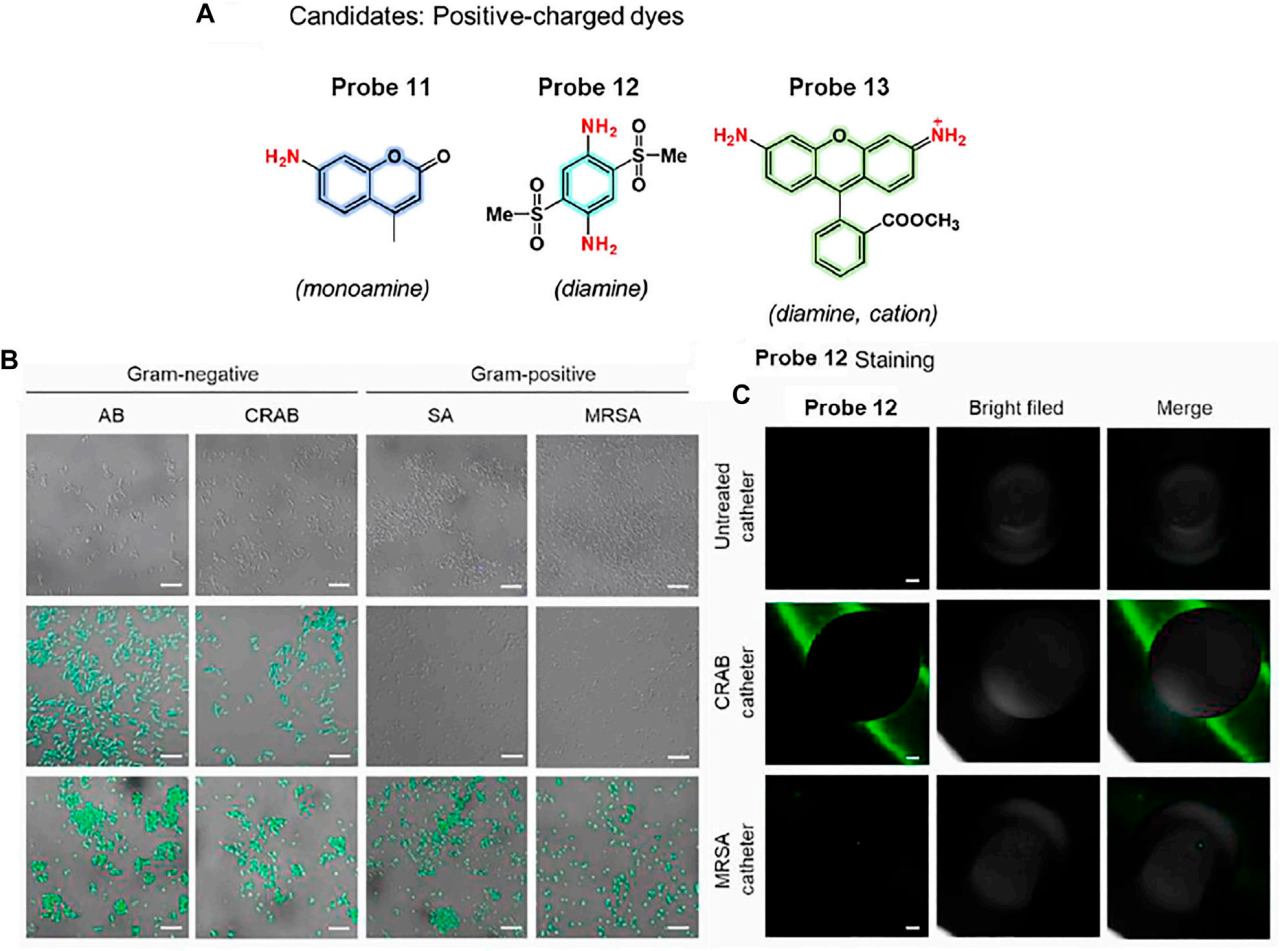
**(A)** Chemical structures of probes 11–13. **(B)** Confocal microscopic images of bacteria incubated with probes 11 (top), 12 (middle), and 13 (bottom). **(C)** CRAB detection of catheter segments using probe 12. Reproduced with permission from Kang et al. (2021). Copyright ^©^ 2020, Elsevier.

Kim et al. proposed a bacterial combinatorial screening method using three fluorophores, probes 11–13 (Figure 7A) (Kang et al., 2021). The three probes have different numbers of positive charges; probe 11 is a monoamine, probe 12 is a diamine, and probe 13 is a cationic diamine. To evaluate the selective imaging ability of this system for Gram-negative and Gram-positive bacteria, the three probes were treated with *Acinetobacter baumannii* (AB), *carbapenem-resistant AB* (CRAB), *Staphylococcus aureus* (SA), and *methicillin-resistant SA* (MRSA). The diamine-based probe 12 exhibited selective fluorescence staining for Gram-negative bacteria, such as AB and CRAB. In contrast, the monoamine-based probe 11 displayed no fluorescence staining, and the cationic diamine-based probe 13 showed fluorescence staining for both Gram-positive and Gram-negative bacteria (Figure 7B). These results indicate that Gram-negative AB and CRAB have a slightly higher negative membrane potential than Gram-positive SA and MRSA, and it was well matched with the electrostatic charge of probe 12. The absorption and emission maxima of probe 12 were recorded at 375 and 515 nm, respectively. In addition, probe 12 can image bacterial biofilms in urinary catheters, which are important for medical applications. Probe 12 showed bright fluorescence in the catheter incubated with Gram-positive CRAB, whereas negligible fluorescence was observed for the control and MRSA-cultured catheters (Figure 7C). These results suggest that this probe can be utilized for Gram-negative bacterial detection and has further applications in various medical fields.

**FIGURE 8:**
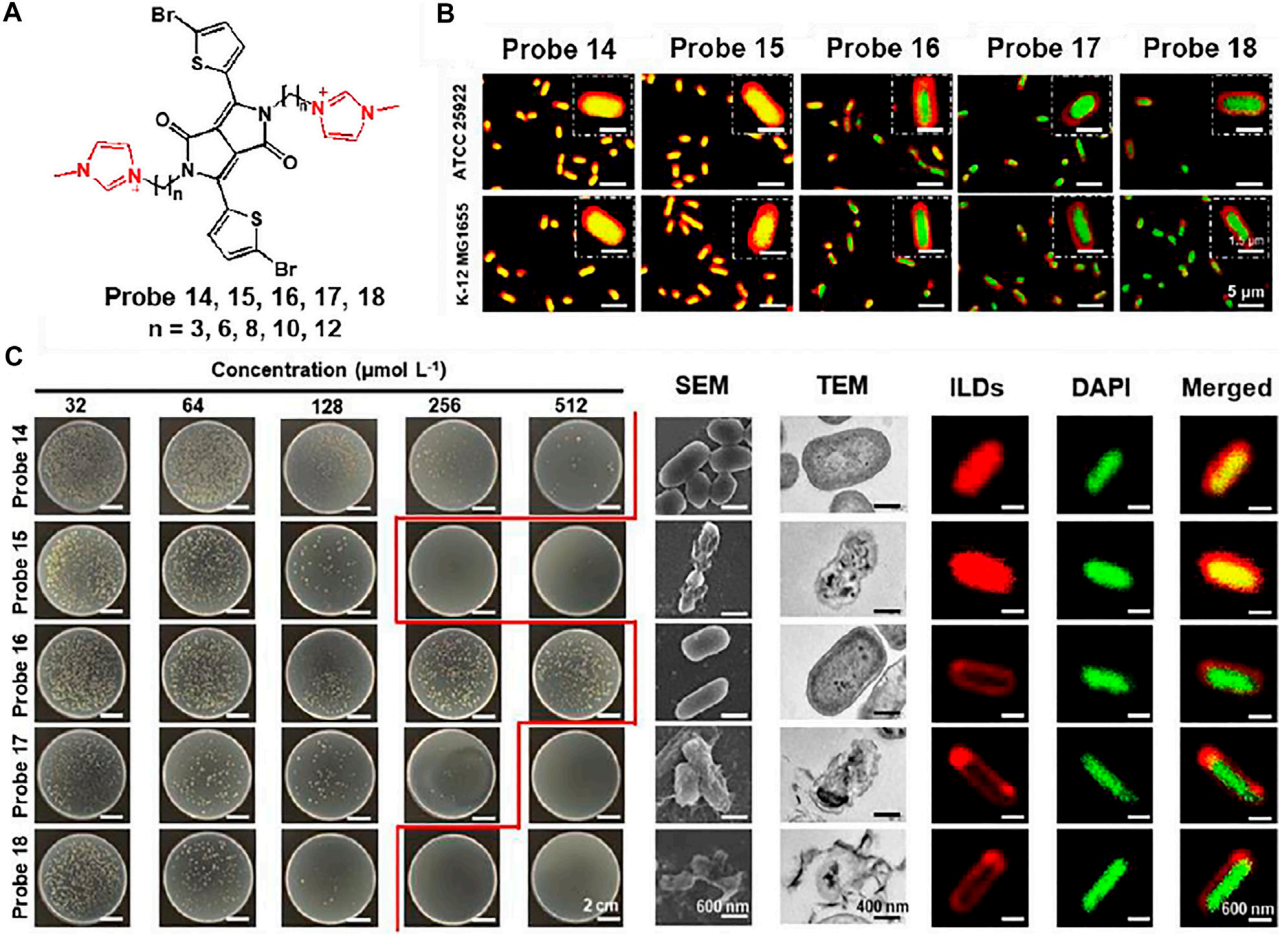
**(A)** Chemical structures of probes 14–18. **(B)** Fluorescent localization of the interactions between probes and *E. coli*. **(C)** Colony of Gram-negative PAO1 incubated with various concentrations of probes, and their SEM, TEM, CLSM images. Reproduced with permission from Zheng et al. (2020). Copyright ^©^ 2020, American Chemical Society.

Xu et al. reported a series of ionic liquid (IL)-based fluorescent probes, probes 14–18 (*n* = 3, 6, 8, 10, 12) for bacterial imaging and demonstration of antibacterial effects (Zheng et al., 2020). Probes are composed of a diketopyrrolopyrrole (DPP) fluorophore and various alkyl chains, showing a correlation between molecular size and antibacterial activity (Figure 8A). To evaluate bacterial penetration ability, the probes were incubated with Gram-negative *E. coli*. Probes 14 and 15 completely entered the bacteria and showed fluorescence, while the fluorescence of probes 16, 17, and 18 was mainly seen in the bacterial membrane (Figure 8B). These results were attributed to the different alkyl chains of the probes: shorter chain-based probes were able to interact with single lobules in the bilayer, and longer chain-based probes were able to interact within the membrane bilayer. This difference in the length of the alkyl chains also affected the antibacterial effect, causing membrane thinning and membrane disorder. The probes were treated with Gram-negative *Pseudomonas aeruginosa* (PAO1) to investigate their antimicrobial effect. Probe 15, with a relatively short chain length, showed a biofilm-thinning ability, leading to a high antibacterial effect. In addition, probes 17 and 18 showed excellent antibacterial activity with membrane-disrupting ability. In contrast, probe 14 had no effect on bacterial membrane damage due to single-layer interactions and probe 15 did not affect the membrane because of its intermediate molecular size (Figure 8C). These results demonstrated that molecular structure and size influence the antimicrobial effect, which may help in the design of more advanced antimicrobial molecular probes.

**FIGURE 9:**
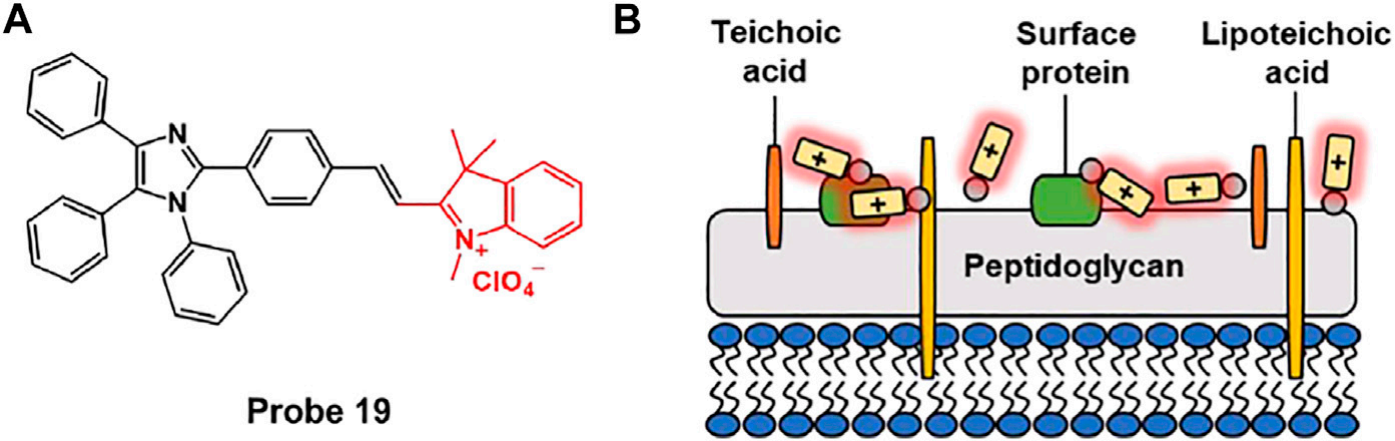
**(A)** Chemical structures of probe 19. **(B)** Schematic illustration of probe 19 for Gram-positive bacteria.

Zeng et al. synthesized probe 19 that can selectively image Gram-positive bacteria based on AIE effect (Figure 9) (Gao et al., 2017). Fluorescence enhancement of probe 19 was observed in aqueous solution using sodium dodecyl sulfate (SDS) as an anionic surfactant. Probe 19 is non-fluorescent in the absence of SDS, but fluorescence at 650 nm sharply increases after the addition of SDS. In addition, formation of spherical nano-agglomerates was confirmed by DLS and SEM. This meant that aggregates were formed between probe and SDS to activate probe’s AIE phenomenon. In bacterial imaging experiments, probe 19 displayed an AIE-based fluorescence in Gram-positive *S. aureus*, in contrast to Gram-negative *E. coli*. It was confirmed that probe 19 containing quaternary ammonium moiety can selectively bind Gram-positive bacteria due to the negatively charged teichoic acid and thick layer of peptidoglycan on the cell wall (Yuan et al., 2014; Gao et al., 2018). This system can be effectively used to differentiate between Gram-negative and Gram-positive bacteria, and can provide a newer strategy for designing AIE-based fluorescent probe for microbial analysis.

## Fluorescent Probes to Detect Bacterial Enzymes

Bactria contain a variety of enzymes that are involved in the biosynthesis of bacterial cell walls, nucleic acids, and metabolites, and are also associated with the bacterial infection process (Bouhss et al., 2008; Egorov et al., 2018). In particular, nitroreductase (NTR) is widely expressed in most bacteria and plays a role in the reduction of aromatic nitro groups to amines. In addition, *E. coli* is reported to be a strain that highly expresses alkaline phosphatase (ALP) involved in dephosphorylation (Kantrowitz, 2011). Antibiotic-resistant bacteria, such as *E. coli/pUC19* and *Mycobacterium tuberculosis*, also express high levels of *β*-lactamases (Shapiro, 2016; Cheng et al., 2018). Caspase-1 is also known to be activated when human cells are infected with bacteria (Sokolovska et al., 2013). Several fluorescent probes have recently been developed that can selectively detect bacteria based on enzymatic reactions.

### Nitroreductase Detection

**FIGURE 10:**
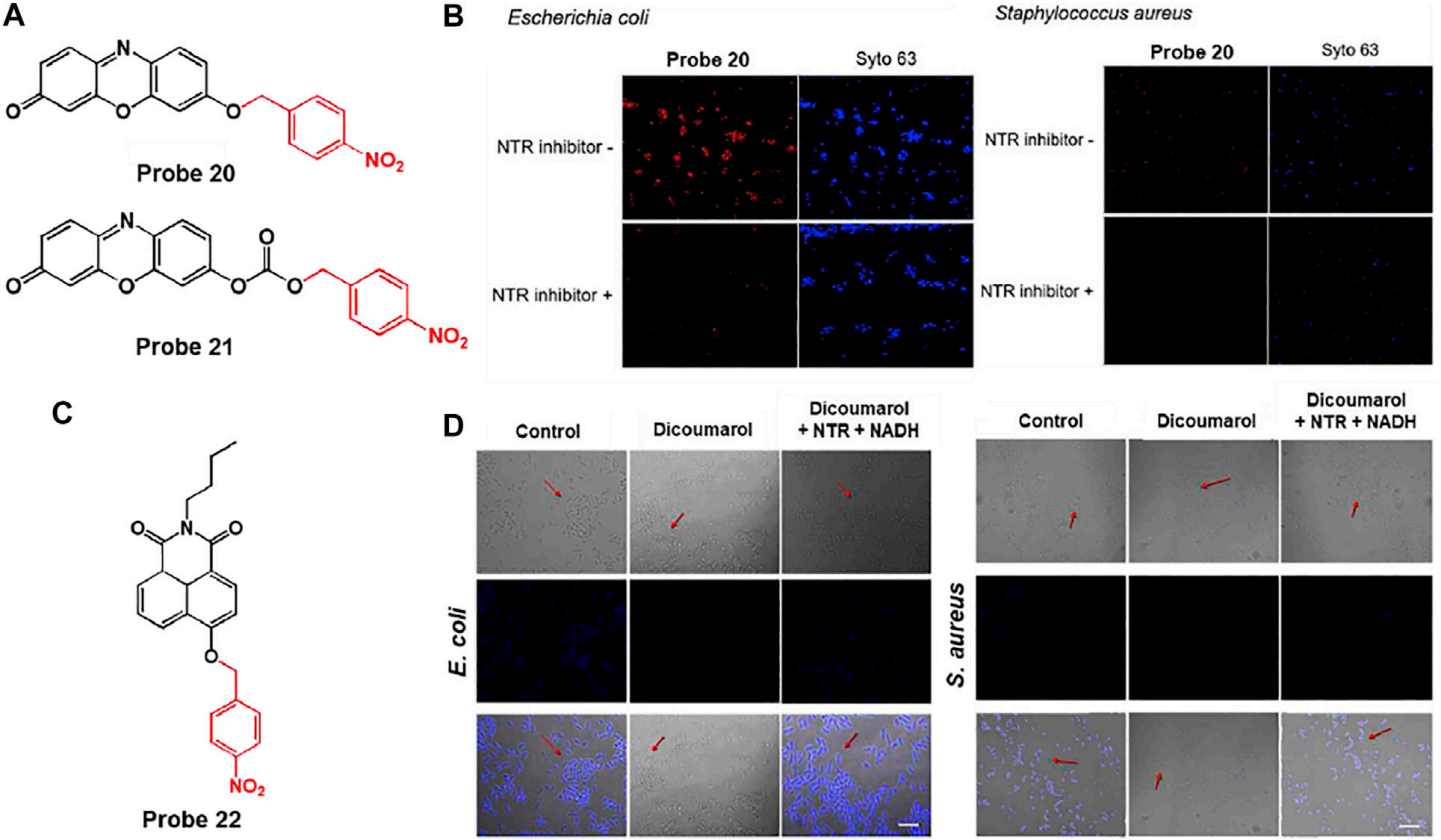
**(A)** Chemical structures of probes 20 and 21. **(B)** Confocal microscopy images of bacteria treated with probe 20 without or with an NTR inhibitor. Syto 63 was used to stain live cells. Reproduced with permission from Yoon et al. (2019). Copyright ^©^ 2019, Elsevier. **(C)** Chemical structure of probe 22. **(D)** Confocal microscopy images of bacteria treated with probe 22 without or with dicoumarol (NTR inhibitor). Reproduced with permission from Zhang et al. (2020a). Copyright ^©^ 2019, Elsevier.

Lee et al. developed a nitroreductase (NTR)-responsive fluorescent probe, probes 20 and 21, composed of resorufin fluorophores linked with *p*-nitrobenzyl units using ether or carbonate groups. In particular, probe 20 can provide a fluorescence turn-on for NTR activity with relatively high stability and selectivity (Figure 10A) (Yoon et al., 2019). In the absence of NTR and NADH, probe 20 showed an absorption band at 450 nm without fluorescence. However, after reaction with NTR in the presence of NADH, a new absorption band emerged at 564 nm with strong fluorescence at 586 nm. Based on these photochemical properties, the NTR detection ability of probe 20 was investigated using *E. coli* and *S. aureus*. Incubation with bacteria and probe 20 displayed a bright red fluorescence. However, pre-incubation with dicoumarol, a known NTR inhibitor, showed very weak fluorescence intensity for bacterial NTR activity (Figure 10B). Moreover, to explore the fluorescence response of the probe in dead cells, the bacteria cell suspensions were heat-treated at 85°C for 10 min. This revealed that the red fluorescence from the probe remarkably decreased in dead cells, which did not synthesize NTR activity. These results demonstrated that probe 20 could display bacterial NTR-mediated fluorescence signals with high selectivity and sensitivity, and also suggest the possibility of its application in the detection of pathogenic bacteria.

Similarly, Sun and coworkers reported the NTR-sensitive fluorescent probe 22, which is comprised of *p*-nitrobenzyl as an NTR reactive moiety and naphthalimide as a signaling fluorophore (Figure 10C) (Zhang et al., 2020a). To NTR in the presence NADH, the nitro group of the *p*-nitrobenzyl moiety was reduced to an amine group and subsequently cleaved from the probe, leading to a fluorescence turn-on. The absorption and emission maximum wavelengths were recorded at 420 and 491 nm, respectively. The detection limit was determined to be 3.4 ng/ml. In the biological experiments, this probe was able to selectively image not only bacteria such as *E. coli* and *S. aureus*, but also hypoxic cancer cells such as HepG2 and MCF-7. This is because the probe showed fluorescence in response to NTR activity, which was confirmed by treatment with dicoumarol, an NTR inhibitor (Kong et al., 2019) (Figure 10D).

**FIGURE 11:**
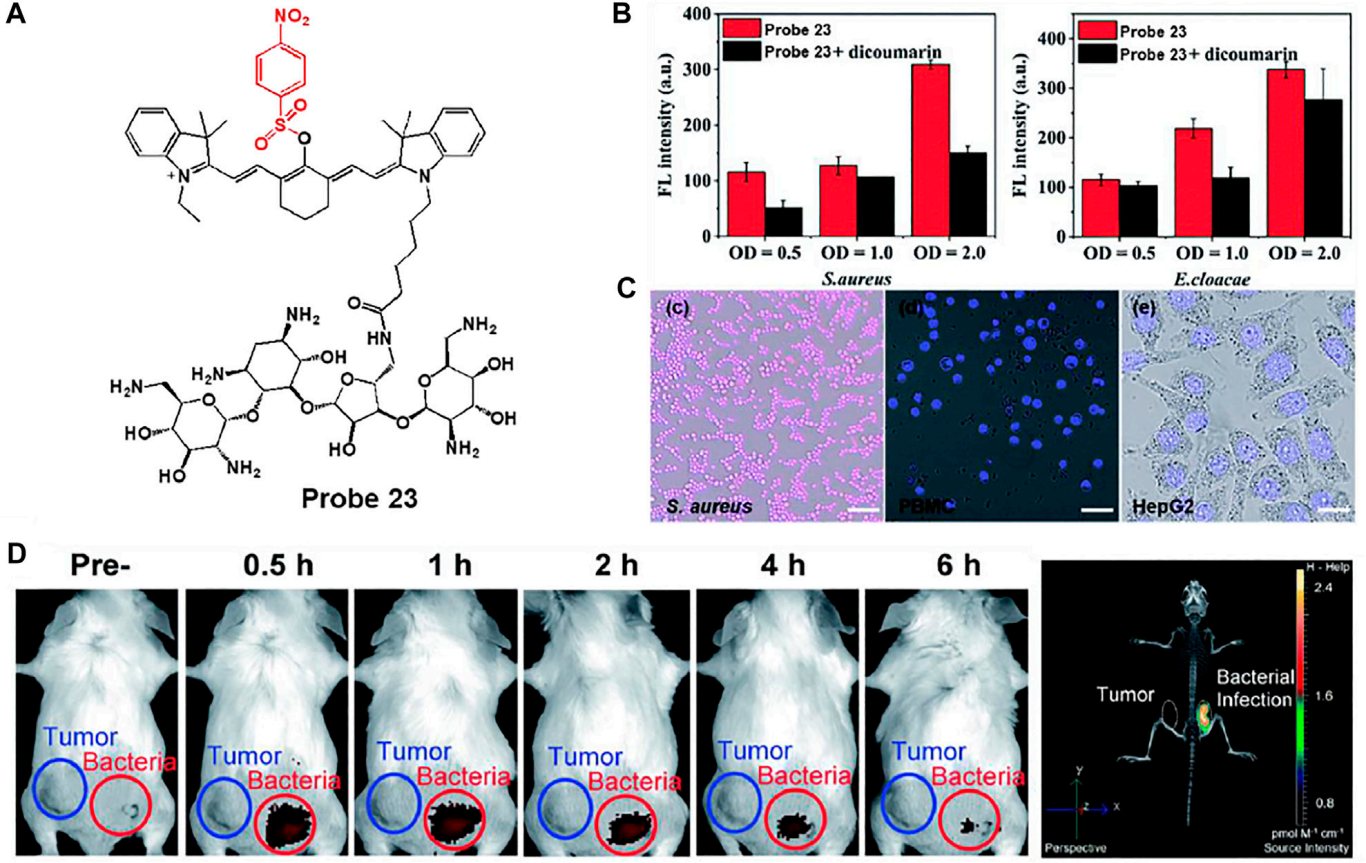
**(A)** Chemical structure of probe 23. **(B)** Quantification of the fluorescence intensity of probe 23 after incubation with *S. aureus* and *E. cloacae* in the presence (black bars) or absence (red bars) of dicoumarin **(C)** Confocal microscopy images of *S. aureus*, PBMC and HepG2 incubated with probe 23. **(D)** Whole-mouse time-dependent images of CT26 tumor-bearing (blue circle) and *S. aureus* infected (red circle) mice before and post i.v. injection of probe 23. Reproduced with permission from Wu et al. (2020). Copyright ^©^ 2020, Royal Society of Chemistry.

Hu et al. developed a near-infrared (NIR) fluorescent probe 23 consisting of cyanine dye attached to a *p*-nitroaromatic group for a rapid response to NTR and neomycin as an aminoglycoside antibiotic for bacterial targeting (Figure 11A) (Wu et al., 2020). Owing to the electron-withdrawing characteristic of the nitro group, the original NIR fluorescence feature of the cyanine dye was quenched. However, in the presence of NTR and NADH, NIR fluorescence was restored by reducing the nitro group to the amine unit. The maximal absorption and fluorescence wavelengths of probe 23 were monitored at 780 and 801 nm, respectively, and the enzyme reaction was completed within 3 min. The limit of detection was assessed as 0.67 ng/ml. To evaluate the detection ability of NTR in bacteria, probe 23 was treated with Gram-positive *S. aureus* and Gram-negative *E. cloacae*. Probe 23 showed a distinct fluorescence increase in both bacteria (Figure 11B). On the other hand, in the presence of dicoumarin, an NTR inhibitor, probe 23 showed reduced fluorescence intensity. Additionally, probe 23 exhibited brighter fluorescence features in bacteria than the human peripheral blood mononuclear cell line (PBMC) and the human hepatoma cell line (HepG2) (Figure 11C). Moreover, in the *in vivo* mouse model, probe 23 showed a strong fluorescence increase at the site of bacterial infection within 30 min, unlike the tumor area (Figure 11D). These results indicate that the probe can be used to understand bacterial infections and related diseases *in vitro* and *in vivo*.

### Alkaline Phosphatase Detection

**FIGURE 12:**
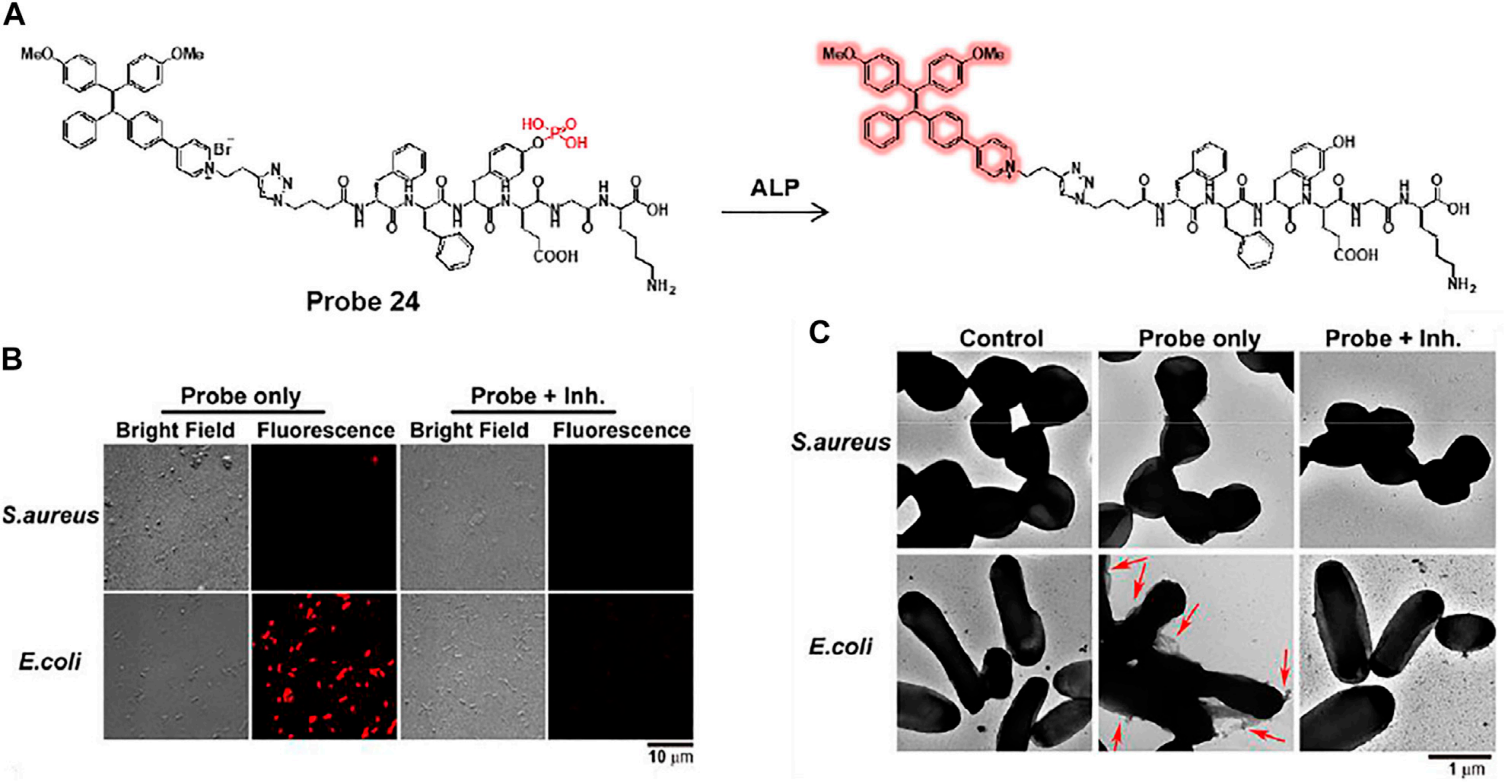
**(A)** Dephosphorylation reaction of probe 24 by ALP. **(B)** Confocal microscopy and **(C)** TEM images of *S. aureus* and *E. coli* after incubation of probe 24 with or without L-phenylalanine, an ALP inhibitor. Red arrows indicate the fibrous structure on the surface of *E. coli*. Reproduced with permission from Zhang et al. (2020b) Copyright ^©^ 2020, American Chemical Society.

In another study, Yang et al. developed an alkaline phosphatase (ALP)-activated fluorescent turn-on AIEgen-peptide conjugate probe 24 (Figure 12A) (Zhang et al., 2020b). Probe 24 can undergo dephosphorylation by bacterial ALP to form an aggregated fibrous structure on the bacterial surface and provide AIE-based fluorescence. Probe 24 showed excellent selectivity and sensitivity for ALP activity, with a detection limit of 6.6 × 10^–3^ U ml^−1^. *E. coli* and *S. aureus* were used to confirm the ALP detection ability of the probe, where *E. coli* is known to be a highly ALP-expressing strain (Kantrowitz, 2011). Bright fluorescence was observed only in *E. coli*, in contrast to *S. aureus* (Figure 12B). In the presence of L-phenylalanine, an ALP inhibitor, the fluorescence was dramatically reduced in *E. coli* (Fernley and Walker, 1970). The TEM image showed that fibrous structures formed on the surface of the probe-treated *E. coli* (Figure 12C). From these results, it was demonstrated that probe 24 can provide a fluorescence turn-on activated by ALP activity and can be used for the selective detection of highly ALP-expressing bacteria.

### *β*-Lactamase Detection

**FIGURE 13:**
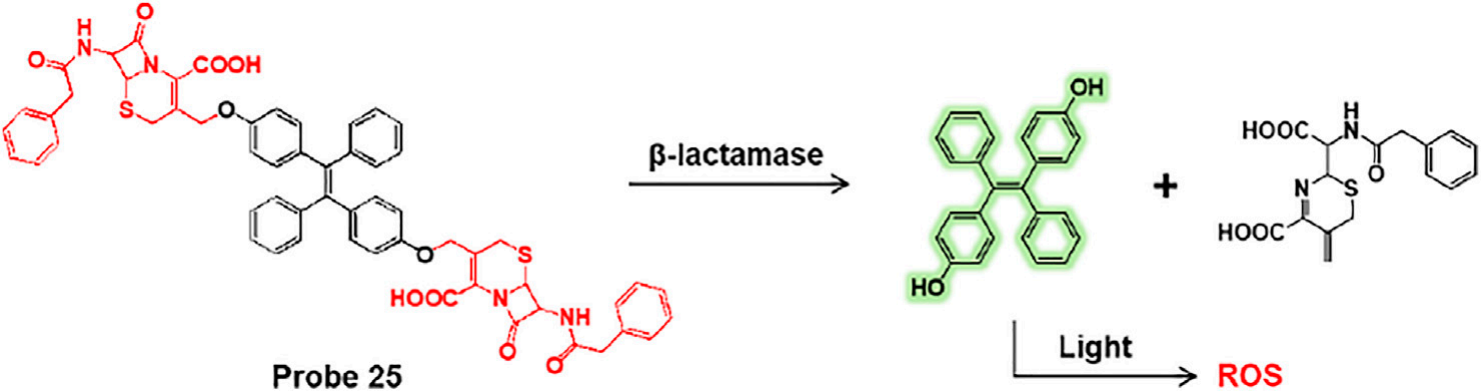
Cleavage reaction of probe 25 by *β*-lactamase.

Li et al. presented tetraphenylethylene-linked cephalosporin (probe 25) for the detection and simultaneous destruction of antibiotic-resistant *E. coli* (Zhao et al., 2020). Probe 25 specifically undergoes a *β*-lactamase-mediated cleavage reaction and gives rise to fluorescence turn-on (Figure 13). Additionally, the cleaved TPE-OH moiety acts as a photosensitizer that destroys bacteria by generating reactive oxygen species (ROS) under light irradiation. The fluorescence responses of probe 25 were investigated using various bacteria captured on the fibers. Among bacteria, probe 25 showed highly effective fluorescence enhancement against *E. coli/pUC19*, an antibiotic-resistant bacterium that expresses high levels of *β*-lactamases compared to other bacteria. The fibrous colored strips also displayed a fluorescence change from blue to green with increasing levels of *E. coli/pUC19*. This fibrous strip offers potential for the real-time detection of antibiotic-resistant bacteria. Meanwhile, SEM images revealed that the probe 25-treated *E. coli/pUC19* exhibited bacterial contraction and fusion by light irradiation in contrast to that in the dark. This demonstrated that probe 25 could selectively detect antibiotic-resistant bacteria expressing *β*-lactamase and selectively destroy antibiotic-resistant bacteria.

**FIGURE 14:**
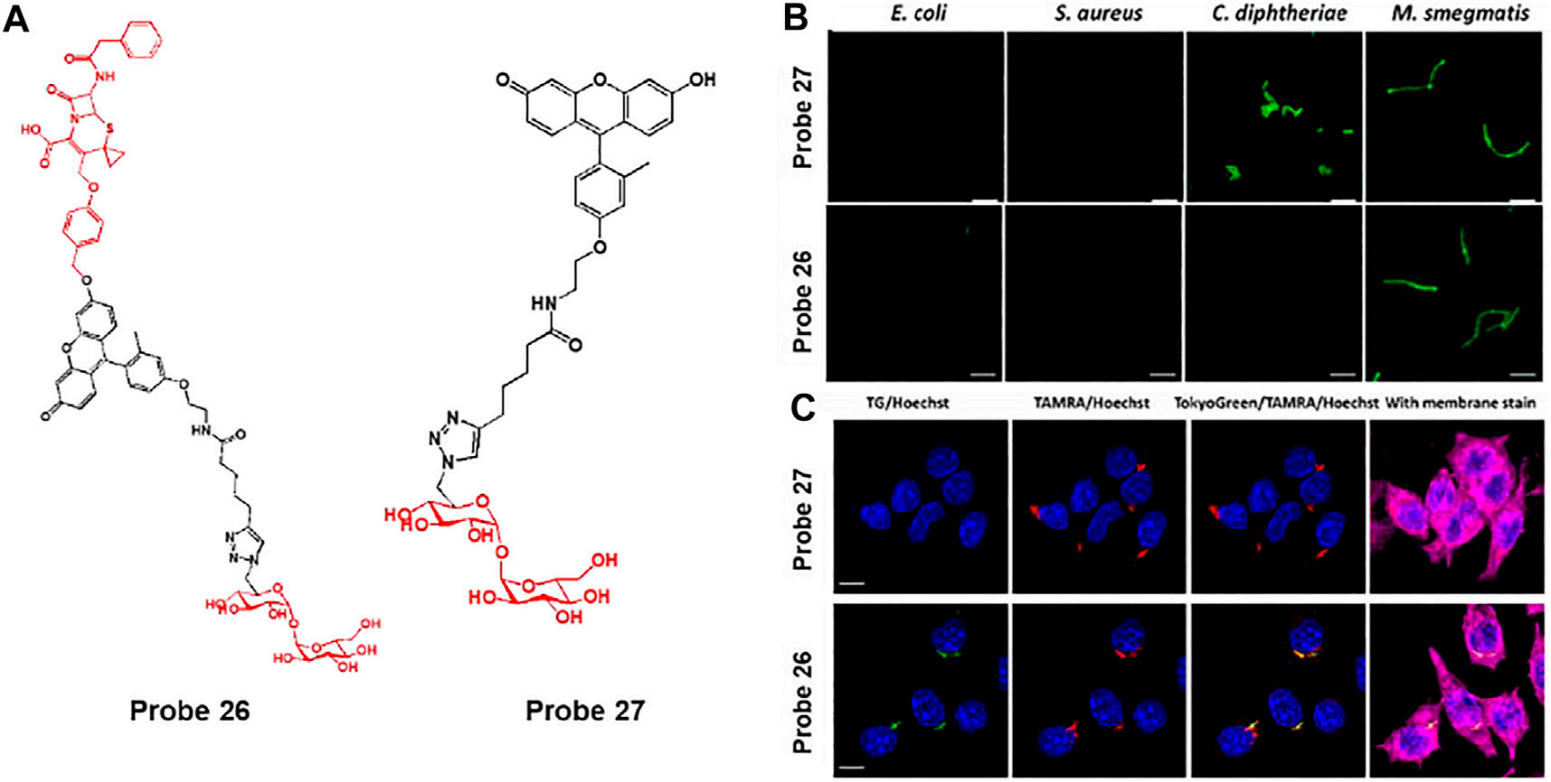
**(A)** Chemical structures of probes 26 and 27. **(B)** Confocal microscopy images of bacteria incubated with 50 μM of probes 27 and 26 in PBS at 37°C for 2 h. Ex = 490 nm/Em = 520 nm. **(C)** Phagocytosed BCG cells were post-labeled by probes 26 and 27 (10 μM) and imaged with a super-resolution structured illuminated microscope. Blue = Hoechst; green = probe; red = TAMRA; deep red = membrane stain. White arrows indicate the asymmetrical labeling of a single BCG. Reproduced with permission from Dai et al. (2020). Copyright ^©^ 2020, American Chemical Society.

In addition, Rao et al. explored cephalosporin as a *β*-lactamase reactive moiety and trehalose as an Ag85s targeting group embedded fluorescent probe 26, which can improve the labeling of phagocytosed *Mycobacterium tuberculosis* (Mtb) and induce fluorescence activation by *β*-lactamase (Figure 14A) (Dai et al., 2020). The lactam ring of cephalosporin is hydrolyzed by *β*-lactamase to produce a highly fluorescent xanthene derivative (probe 27). Subsequently, probe 27 labels the bacterial cell wall *via* the Ag85s-mediated trehalose pathway (Belisle et al., 1997). In confocal imaging, probes 26 and 27 were used with various bacteria such as *E. coli*, *S. aureus*, *C. diphtheriae*, and *M. smegmatis* (Figure 14B). Probe 26 showed strong fluorescence features only for *M. smegmatis*, while probe 27 exhibited strong green fluorescence for *C. diphtheriae* and *M. smegmatis*. *C. diphtheriae* and *M. smegmatis* belong to the Actinobacteria phylum with a mAG layer, but only *M. smegmatis* naturally expresses *β*-lactamase (Flores et al., 2005). To confirm whether probe 26 can image Bacillus Calmette–Guérin (BCG) cells within macrophages, TAMRA-Tre-pre-labeled BCG cells were incubated with macrophages and then processed with probes 26 and 27 (Figure 14C) (Rodriguez-Rivera et al., 2018). The probe 26 treated cells showed green fluorescence in BCG cells and overlaid with the red fluorescence of TAMRA-Tre. On the other hand, probe 27 showed almost no fluorescence under the same conditions. These results demonstrate that probe 26 could visualize BCG cells in macrophages.

### Caspase-1 Detection

**FIGURE 15:**
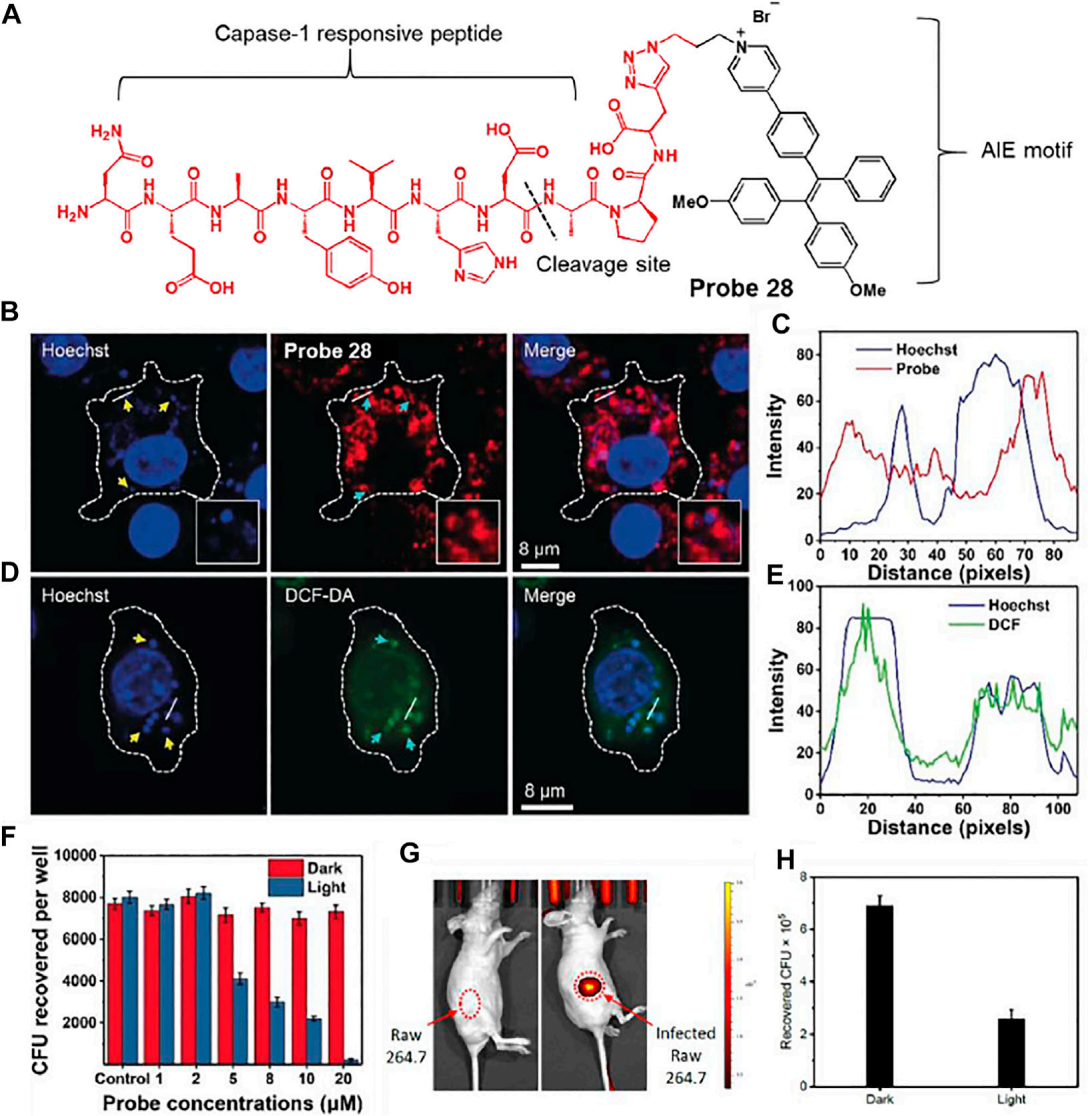
**(A)** Chemical structure of probe 28. **(B)** Confocal microscopy images showing the localization of probe 28 after 60 min incubation in Raw 264.7 macrophages infected with *S. aureus*. **(C)** Fluorescence profiles of probe 28 (red) and bacterial DNA staining Hoechst dye (blue) across the phagosomes containing *S. aureus* (from the white straight lines in the confocal images). **(D)** Confocal microscopy images of ROS inside the macrophages using DCF-DA after bacterial infection in the presence of probe 28. **(E)** Fluorescence profiles of DCF-DA (green) and bacterial DNA staining Hoechst dye (blue) across the phagosomes containing *S. aureus* (from the white straight lines in the confocal images). Yellow arrows indicated *S. aureus*, while cyan arrows did phagosomes containing *S. aureus*. **(F)** Intracellular survival of *S. aureus* in the Raw 264.7 macrophages at different concentrations of probe 28 without and with light irradiation for 10 min at 40 mW cm^−2^. **(G)** *In vivo* fluorescence images of intracellular bacteria-bearing mice after i.v. injection of probe 28. **(H)** CFU of *S. aureus* monitored from the infected regions subjected to dark or light irradiation. Reproduced with permission from Qi et al. (2019). Copyright ^©^ 2019, Wiley-VCH Verlag GmbH & Co. KGaA, Weinheim.

Liu et al. developed a caspase-1 responsive peptide linked aggregation-induced emission luminogen (AIEgen) probe 28 to detect and destroy intracellular bacteria (Figure 15A) (Qi et al., 2019). Since caspase-1 is activated when macrophages are infected with bacteria, the detection of caspase-1 is of great interest (Sokolovska et al., 2013). It was proposed that probe 28 can be cleaved by caspase-1 in bacteria-infected macrophages, and the resulting AIEgen residues are self-assembled and accumulate with fluorescence-On specifically in bacteria containing phagosomes. Probe 28 showed selective fluorescence enhancement in infected macrophage lysates treated with caspase-1, unlike other enzymes. In confocal imaging, AIE-based fluorescence from probe 28 was observed in macrophages infected with *S. aureus* and *E. coli*, in contrast to macrophages without bacterial infection. To determine the localization of residues, Hoechst dye and probe 28 were co-incubated with macrophages infected by *S. aureus* (Figures 15B,C). The DNA of both macrophages and bacterial phagosomes was labeled with Hoechst dye (blue). In addition, since AIEgen residues act as a photosensitizer under light irradiation to generate reactive oxygen species (ROS), they exhibit green fluorescence images by DCF-DA, an ROS indicator (Figures 15D,E) (Wang et al., 2019). The green image overlaps with the bacterial phagosomes (blue). Based on these results, the AIEgen residues seemed to accumulate on the phagosome containing *S. aureus* and generated ROS under light irradiation. To investigate whether the probe could destroy the bacteria from macrophages, macrophages were infected with *S. aureus* and treated with different concentrations of probe in the dark and under light irradiation. Survival of intracellular bacteria was determined by counting the colony forming units (CFU) (Figure 15F). This showed a concentration-dependent reduction in intracellular *S. aureus* under light irradiation. It also showed that mice bearing bacteria-infected macrophages were treated with probe 28 to selectively allow for *in vivo* fluorescence imaging of the infected site (Figure 15G). The infected region was subjected to light irradiation for 10 min, and the CFU was markedly reduced compared to that in the non-irradiated group (Figure 15H). This study presented a platform to combine the detection of bacterial infections with photodynamic therapy.

## Chemically Fluorescent Labelling of Bacteria

Bacterial cell walls are composed of peptidoglycan (PG) and retain their shape and viability. PG is a macromolecular polymer network containing alternating *N*-acetylmuramic acid (NAM) and *N*-acetylglucosamine (NAG) units cross-linked by D-amino acid (DAA)-containing peptide chains (Kuru et al., 2012; Kuru et al., 2015). Currently, a number of fluorescent probes for bacterial labeling have been reported that utilize D-amino acid (DAA). Amino acids typically exist in the L-form in mammalian cells (Aliashkevich et al., 2018), and thus, DAAs act as useful tools for bacterial targeting. DAA participated in PG synthesis, and the linked fluorophores emitted fluorescence in a congested PG environment by aggregation-induced emission (AIE) or twisted intramolecular charge transfer (TICT) effects.

**FIGURE 16:**
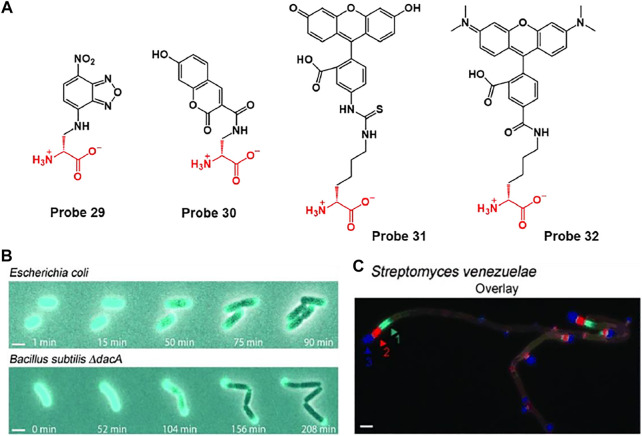
**(A)** Chemical structures of probes 29–32. FDAAs label diverse bacterial growth patterns. **(B)** Time-lapse microscopy of HADA-labeled *E. coli* and *B. subtilis ΔdacA* cells imaged during growth on LB agarose pads (LB = Luria Bertani). **(C)** Triple labeling of *S. venezuelae* with probe 29 (green), 30 (blue), and 32 (red). Arrows in the triple-labeling panels indicate the sequence of labeling. Reproduced with permission from Kuru et al. (2012). Copyright ^©^ 2012 WILEY-VCH Verlag GmbH & Co. KGaA, Weinheim.

For example, VanNieuwenhze et al. developed D-alanine-containing probes 29 and 30 using hydroxycoumarin and nitrobenzofurazan as fluorophores and D-lysine-containing probes 31 and 32 using fluorescein and carboxytetramethylrhodamine (TAMRA) as fluorophores (Figure 16A) (Kuru et al., 2012). Each probe 29–32 exhibited maximum fluorescence emissions at 450, 538, 515 and 565 nm, respectively. D-amino acids (DAA), such as D-alanine and D-lysine, participate in peptidoglycan (PG) synthesis and are incorporated into the PG peptide chain. In addition, these probes can be incorporated into the cell wall by an enzyme-catalyzed periplasmic exchange reaction to clearly mark the active PG synthesis site. Using this mechanism, probes could obtain the time-lapse images of probe 30-treated *E. coli* and *B. subtilis ΔdacA* cells during growth on Luria Bertani (LB) agarose pads (Figure 16B). As time goes by, fluorescence signal of probe 30 was retained in the bacterial polar cap, but gradually disappeared from the lateral walls. This signal change is in good agreement with previous reports that the cell wall grows along the length of the lateral walls (Mobley et al., 1984). By using the different fluorescence colors of probes, the position and degree of PG synthesis activity during each labeling period were indicated, thereby confirming the chronological explanation of the movement of bacterial PG synthesis over time (Figure 16C).

**FIGURE 17:**
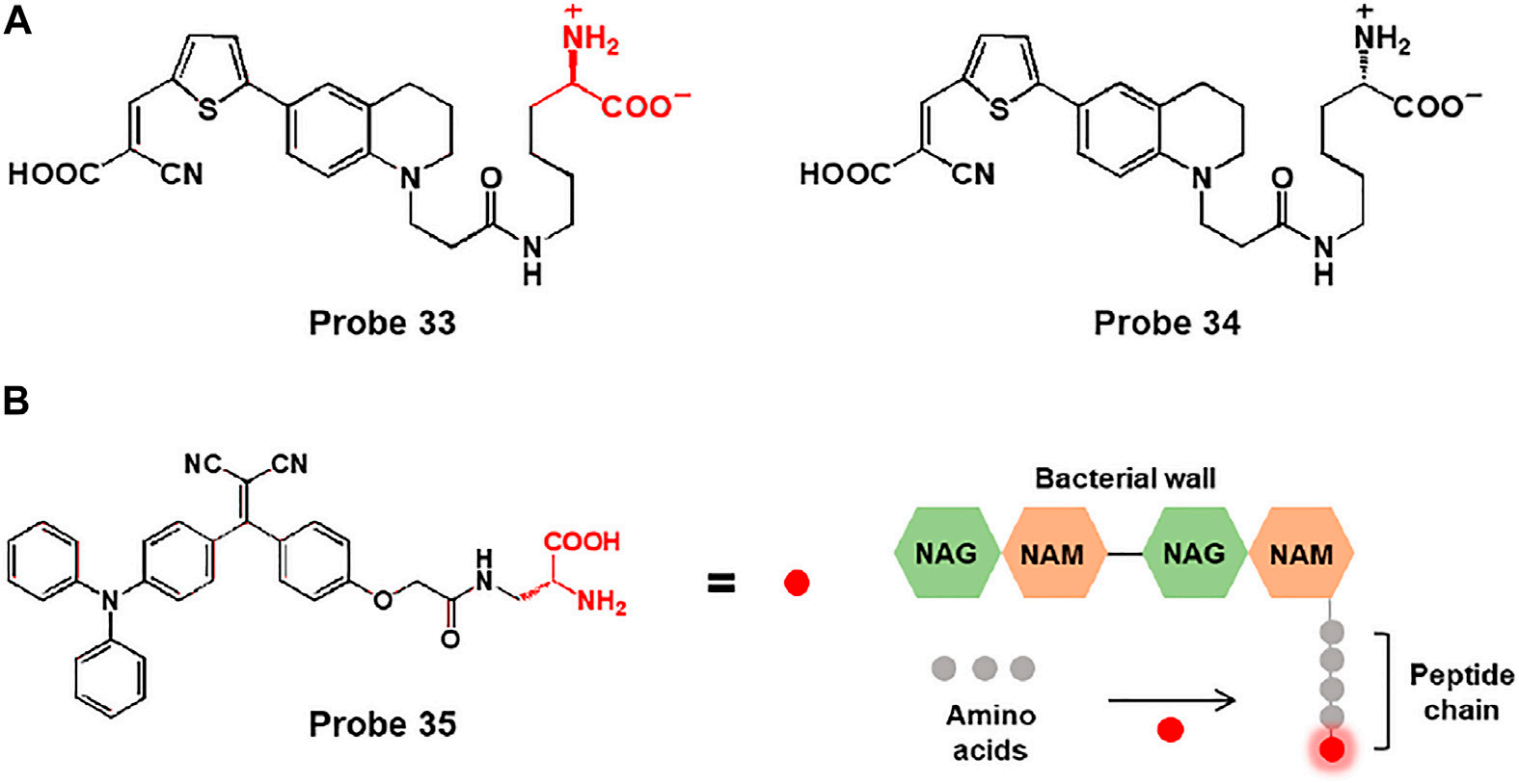
**(A)** Chemical structures of probes 33 and 34. **(B)** Chemical structure of probe 35 and the schematic of the bacterial wall labelling with the D-Ala moiety.

Furthermore, they were developed a rotor-fluorogenic D-amino acid-based probe 33 for real-time monitoring of peptidoglycan biosynthesis, in which the cyanoacrylic acid subunit, which acts as a TICT fluorophore, is linked to D-lysine (Figure 17A) (Hsu et al., 2019). During peptidoglycan synthesis, D-lysine of probe 33 integrated into peptidoglycan peptide chains and exhibited fluorescence emission at 640 nm with an excitation wavelength of 470 nm. In biological studies, probe 33 was used to image bacterial cells through peptidoglycan labeling. In comparison with fluorogenic D-amino acid (FDAA), HADA (7-hydroxycoumarin-3-carboxylic acid-amino-D-alanine), which showed strong background fluorescence, probe 33 could be used without washing steps due to the “off-on” fluorescence responses of RfDAAs. In addition, probe 34 (L-enantiomer of probe 33) showed no fluorescence signal, which indicates that fluorescence emission is caused by D-amino acids involved in peptidoglycan transpeptidation. Moreover, probe 33 could visualize peptidoglycan synthesis in bacterial cells in real time. Newly formed cell branches and division septa are represented by red fluorescence. Furthermore, the fluorescence responses of probe 33 were investigated following treatment with various antibiotics. Cefoxitin is a *β*-lactam antibiotic drug that inhibits transpeptidase activity, resulting in blocked peptidoglycan synthesis, whereas chloramphenicol, a ribosome peptidyl transferase inhibitor, showed no inhibitory effect. As expected, no fluorescence response was observed in the presence of cefoxitin, whereas an increase was observed in the presence of chloramphenicol. Therefore, probe 33 could image peptidoglycan biosynthesis and be applied to real-time assays of transpeptidase activity.

In another study, Liu and coworkers reported a bacteria-labelling theranostic probe 35 consisting of an AIE-based fluorescent photosensitizer and D-alanine (D-Ala) (Figure 17B) (Mao et al., 2020). When probe 35 reached bacteria, the D-Ala moiety bonded to the bacterial wall, and the probe showed fluorescence emission due to the AIE effect. In biological applications, with good water solubility and small size, probe 35 easily penetrated the *MRSA*-infected RAW 264.7 cells and biofilms and bonded to the bacterial wall to produce fluorescence emission. Moreover, the probe could eliminate bacteria through ^1^O_2_ generation induced by light irradiation. Bacterial viability of *MRSA*-infected cells and biofilms was significantly decreased under light irradiation. For *in vivo* imaging studies, mice were injected with *S. aureus* WH^GFP^ into the right flank skin. After intravenous injection of probe 35, the images of the infected mice showed enhanced fluorescent signals. In addition, the antibacterial ability of probe 35 was examined in infected skin tissues of mice. The colony-forming units (CFUs) of probe-treated and light-irradiated skin diminished, and H&E staining images showed less inflammatory cell infiltration compared to untreated tissue. Thus, probe 35 could act as a bacteria-targeting theranostic probe for bacterial diagnosis and photodynamic therapy *in vitro* and *in vivo*.

## Other Fluorescent Detection in Bacteria

### Fluorescent Probe for Hg (II) Detection in Bacterial Environment

Mercury ions are highly toxic chemicals that accumulate in tissues and organs, causing various diseases such as kidney failure and nervous system disorders (Martín-Yerga et al., 2013). In real-world applications of Hg (II) sensors, analytes are usually polluted with bacteria. It has been reported that Hg^2+^ can be converted to Me-Hg^+^ in some bacteria (Parks et al., 2013), and fluorescence detection of bacterial Hg^2+^ will help to understand this conversion and its mechanism.

**FIGURE 18:**
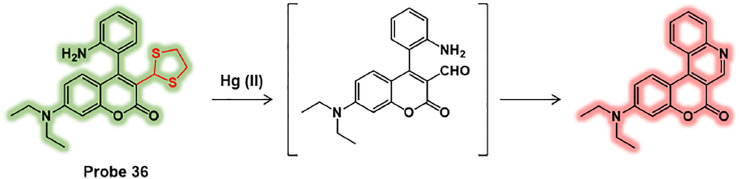
Chemical structure of probe 36 and Hg (II) sensing mechanism.

For instance, Yu et al. reported a ratiometric fluorescent probe for Hg (II), probe 36, consisting of coumarin derivative fluorophore with *ortho*-2-aminophenyl group and 1,3-dithiolane moiety (Figure 18) (Pan et al., 2018). Towards Hg (II), the 1,3-dithiolane group of probe 36 is transformed to formyl group and undergoes an internal cyclization. This Hg (II)-selective response of probe resulted in a ratiometric fluorescence change manner. The detection limit was calculated to be 27 nM of Hg (II). In biological applications, probe 36 has been successfully applied to *E. coli*. As the concentration of Hg (II) increases in *E. coli*, the fluorescence intensity of red channel increased while the signal of green channel was diminished. This research demonstrated that probe 36 can trace Hg (II) in bacteria and it would be an ideal tool to explore the biological transformation of mercury ions in bacteria.

**FIGURE 19:**
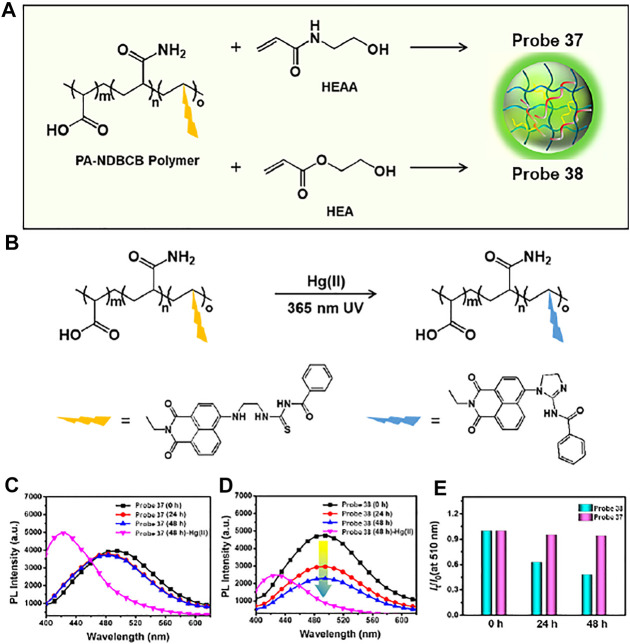
**(A)** Hydrogel components of probes 37 and 38. **(B)** The sensing mechanism of the probes to Hg (II) ion. **(C**,**D)** Time-dependent change of fluorescence spectra between probes 37 and 38 for *E. coli* coculture. **(E)** Emission intensity ratio I_425_/I_510_ of probes 37 and 38 for 0–48 h *E. coli* coculture. Reproduced with permission from Zhang et al. (2019). Copyright ^©^ 2019, American Chemical Society.

Yang and coworkers developed a Hg (II)-selective semi-interpenetrating polymer network (IPN) fluorescent hydrogel, probe 37, which was composed of a hydrophilic poly(N-(2-hydroxyethyl)acrylamide) (polyHEAA) network and interpenetrated fluorescent copolymer PA-NDBCB (Figure 19A) (Zhang et al., 2019). In the absence of Hg (II) ions, probe 37 emitted a green fluorescence at 510 nm. However, upon exposure to Hg (II) ions, the desulfurization/cyclization reaction of the naphthalimide derivative was induced to show blue-shifted fluorescence at 425 nm (λ_ex_ = 365 nm) (Figure 19B). The detection limit of probe 37 for Hg (II) ions was found to be 0.067 µM. In addition, the fluorescence response of probe 37 in a bacteria-laden environment was examined. A semi-IPN fluorescent poly(2-hydroxyethyl acrylate) (polyHEA) hydrogel, probe 38, was synthesized for comparison. PolyHEAA is an antifouling material that prevents bacterial adhesion. Therefore, probe 37 maintained stable fluorescent emission after 48 h coculture in *E. coli*, while probe 38 exhibited reduced fluorescence intensity (Figures 19C–E). Moreover, probe 37 showed enhanced fluorescence emission for Hg (II) ions in polluted water and food samples. These results suggest that probe 37 could be used for the long-term ratiometric detection of Hg (II) ions in bacterial environments.

### Fluorescent Probe for Chloride Detection

Chloride is an essential ion involved in various homeostatic processes and electrical activities (Zajac et al., 2020). Especially, in some marine bacteria, chloride ion-pumping rhodopsin (ClR) uses light energy to actively transport Cl^−^ into cells (Inoue et al., 2014). Additionally, some studies have reported that Cl^−^ is essential for bacterial growth and is important for balancing external salt concentrations (Roeßler et al., 2003). However, it is largely unknown whether chloride is important for the physiology of the bacterial cell. Therefore, it is important to develop fluorescent probe that have valid chloride-binding sites.

**FIGURE 20:**
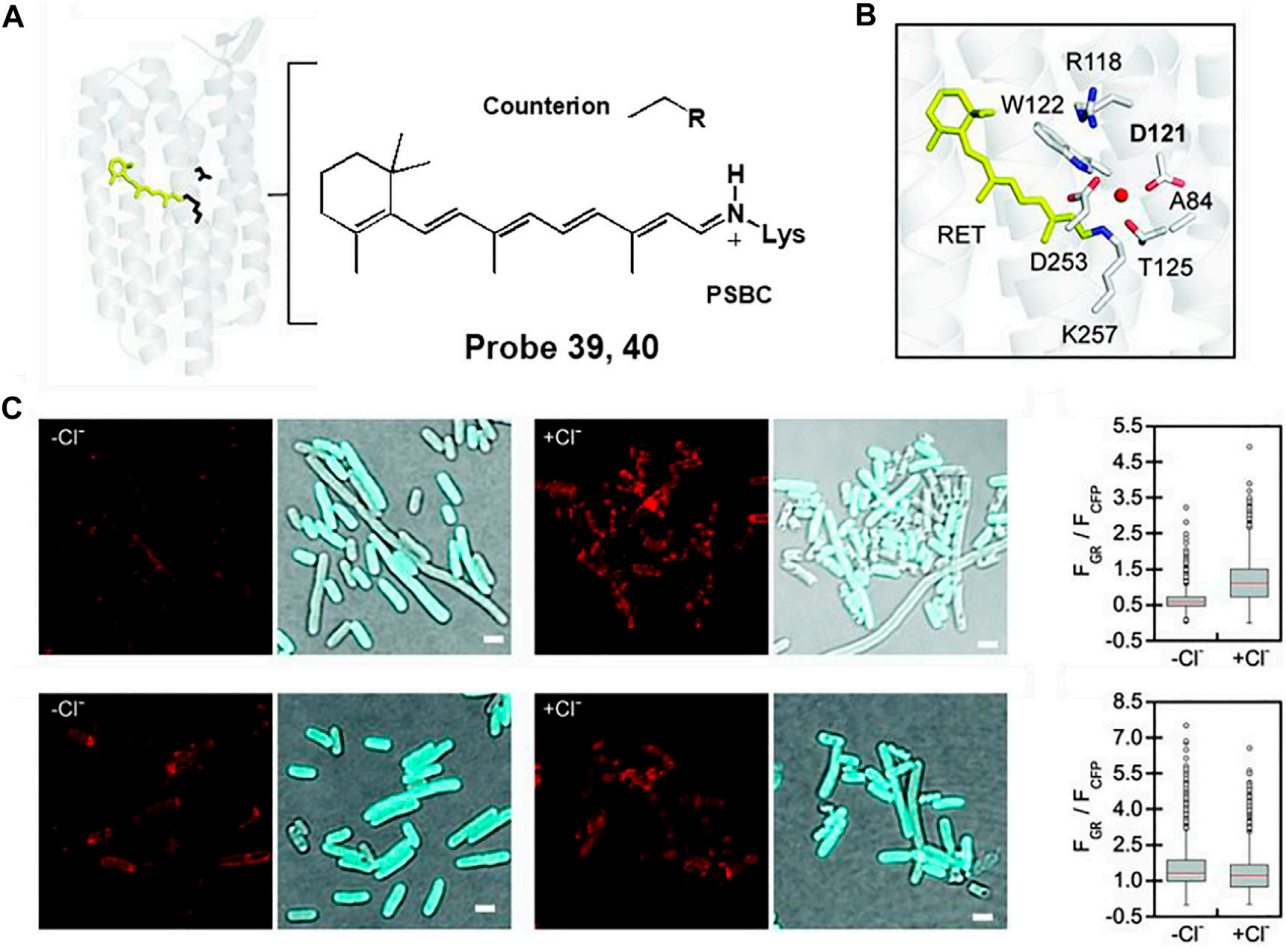
**(A)** Chemical structure of rhodopsin. **(B)** The fluorescent proton pumping rhodopsin from *Gloeobacter violaceus* (wtGR). **(C)** Fluorescence images of probes 39 (top) and 40 (bottom) in the absence and presence of chloride ion. Reproduced with permission from Tutol et al. (2021). Copyright ^©^ 2021, Royal Society of Chemistry.

For example, Dodani et al. reported a chloride-sensitive fluorescent probe 39 comprising a counterion-mutated rhodopsin (GR1) fused to a cyan fluorescent protein (CFP) (Tutol et al., 2021). Rhodopsin is a transmembrane of chloride ion pump composed of a retinylidene Schiff base chromophore (SBC) bonded to seven α-helices (Figure 20A). By mutating the counterion position from aspartate (D121) to valine (D121V), the fluorescent proton pumping rhodopsin, wtGR, transformed into the fluorescent chloride sensor, GR1 (Figure 20B). Fluorescence responses were observed in live *E. coli* cells expressing probes 39 or 40 (Figure 20C). In the absence of chloride, probes 39 and 40 showed chloride-insensitive CFP fluorescence at 425–560 nm (λ_ex_ = 390 nm). In the presence of chloride, chloride binds to GR1 and increases the pK_a_ of the SBC, resulting in a shift of equilibrium towards the protonated state (PSBC). Therefore, probe 39-treated *E. coli* displayed increased fluorescence emission of GR1 at 600–800 nm (λ_ex_ = 530 nm), whereas the fluorescence response of probe 40-treated *E. coli* showed negligible changes. The detection limit of probe 39 was determined to be 12.5 mM. In addition, the fluorescence response of probe 39 could be reversibly changed by sodium acetate buffer or sodium gluconate. These results confirmed that probe 39 was capable of detecting dynamic changes in chloride ions in live *E. coli* cells.

## Conclusion

This review focuses on an overview of recent advances in the development of fluorescence-based dyes capable of detecting bacteria. Several organic fluorescent probes have been actively studied to overcome the limitations of conventional detection methods. These probes can generate “off-on” fluorescence through bacterial-specific reactions and interactions, allowing real-time fluorescent imaging of bacteria and quantitative analysis of bacteria *in vitro* or *in vivo*. Bacterial targeting strategies include specific interactions with cell wall components such as lipopolysaccharide (LPS), reactions of enzymes such as nitroreductase (NTR), alkaline phosphatase (ALP), *β*-lactamases, and caspase-1, and include peptidoglycan (PG) synthesis. Other miscellaneous detections in bacteria are also outlined. In particular, some probes can differentiate between Gram-negative and Gram-positive bacteria or selectively detect antibiotic-resistant bacteria. In addition, studies on the development of theranostic probes that exert photodynamic antimicrobial effects concurrently with bacterial analysis are of considerable interest.

Although many organic fluorescent probes have been developed for bacterial detection and related applications, further advances are required. In fact, there is a need to develop a fluorescent probe that can accurately and quantitatively analyze trace bacteria in the body without any other interference. In addition, developing fluorescent probes that are sensitive to specific bacteria could provide a key strategy for diagnosing bacterial infections and developing antibacterial agents. We hope that this review will help advance the development of fluorescent probes for effective bacterial detection and the design of biological validation models.
